# Effect of glomerular filtration rate impairment on diagnostic performance of neutrophil gelatinase-associated lipocalin and B-type natriuretic peptide as markers of acute cardiac and renal failure in chronic kidney disease patients

**DOI:** 10.1186/cc13752

**Published:** 2014-02-28

**Authors:** Carlo Donadio

**Affiliations:** 1 Department of Clinical and Experimental Medicine, Division of Nephrology, University of Pisa, Via Roma 67, 56127 Pisa, Italy

## Abstract

**Introduction:**

Cardio-renal syndromes are characterized by the impairment of cardiac and renal functions. Plasma and urinary neutrophil gelatinase-associated lipocalin (NGAL), and plasma B-type natriuretic peptide (BNP) are markers of acute kidney injury (AKI) and heart failure (HF), respectively.

The aim of this study was to assess the effect of the reduction of glomerular filtration rate (GFR) on plasma BNP and on plasma and urinary NGAL concentration**s** in stable chronic kidney disease (CKD) patients at different functional stages.

**Methods:**

GFR (^99m^Tc-DTPA), plasma BNP, and plasma and urinary concentrations of NGAL were measured in 310 clinically stable CKD patients, at functional stages from 1 to 5. Serum and urinary low-molecular-weight proteins cystatin C and β2-microglobulin, and urinary tubular enzymes were measured for comparison. Plasma BNP, NGAL, cystatin C and β2-microglobulin were measured also in 31 maintenance hemodialysis patients.

**Results:**

Plasma NGAL increased with the reduction of GFR in CKD patients from stage 2. In the different CKD stages modest differences were found for BNP values. Urinary NGAL increased slightly but significantly in patients at CKD stages 4 and 5, similarly to urinary cystatin C and β2-microglobulin. In maintenance hemodialysis patients, plasma NGAL and BNP were markedly increased, and high-flux hemodialysis significantly decreased their plasma concentrations.

**Conclusions:**

Plasma NGAL increases markedly with the reduction in GFR, generating a very high number of false positive diagnoses of AKI in stable CKD patients. The grade of GFR impairment and the cause of kidney disease have a lower effect on urinary NGAL and on plasma BNP. In any case, specific reference values of NGAL and BNP should be used in chronic kidney disease patients, according to their functional stage, when assessing acute kidney injury, heart failure, and cardio-renal syndromes in patients with impaired GFR.

## Introduction

Acute or chronic impairment of both cardiac and renal functions characterize cardio-renal syndromes [1]. Neutrophil gelatinase-associated lipocalin (NGAL), either in plasma or urine, is a marker of acute kidney injury (AKI), while increased plasma levels of B-type natriuretic peptide (BNP) suggest heart failure. Renal impairment in heart failure patients is characterized by decreased glomerular filtration rate (GFR) and increased urinary excretion of albumin and NGAL [2]. Plasma NGAL, which correlates with AKI severity [3], is also a strong predictor of adverse outcomes in acute heart failure patients [4]. These data suggest that renal damage has a role in determining the prognosis of acute heart failure patients [5]. In patients with heart failure and chronic kidney disease (CKD), plasma BNP increases progressively with the reduction of renal function [6]. Plasma BNP also increases, independently from heart failure, in CKD patients with impaired GFR, due to reduction of its renal clearance [7,8]. Only few data are available on the reference ranges of plasma and urinary NGAL in normal subjects [9]. In any case, whether the accuracy of plasma and urinary NGAL as indicators of AKI is affected by the impairment of GFR remains undefined [10]. This information is particularly relevant due to the increase of AKI in CKD patients; that is, acute-on-chronic kidney injury.

The aim of this study was to assess the effect of the reduction of GFR on plasma BNP and plasma and urinary NGAL and, for comparison, on serum and urinary concentrations of low-molecular-weight proteins (LMWPs) and urinary activity of tubular enzymes in stable CKD patients at different functional stages.

## Materials and methods

### Patient recruitment and selection

CKD patients were referred to the laboratory of the Nephrology Division of the Department of Internal Medicine of Pisa University for the assessment of GFR, from January 2007 to June 2009. The majority of patients (≈65%) came from outpatient clinics, the remainder were in-hospital patients. CKD had been diagnosed according to National Kidney Foundation K-DOQI guidelines [11]. Inclusion criterion were age >18 years and diagnosis of clinically stable CKD at any stage. Potential living kidney donors, post-donation kidney donors and renal transplant recipients were also referred for the measurement of GFR. In donors, measurements were performed between 3 and 6 months post nephrectomy. Exclusion criteria were AKI, primary tubular diseases, and recent administration of potentially nephrotoxic drugs. AKI was excluded on the basis of clinical history, reported constancy of urinary output and of serum creatinine levels the week before GFR measurement.

The study was approved by the Institutional Ethical Committee of Azienda Ospedaliero-Universitaria Pisana and conducted in accordance with guidelines of Helsinki declaration and with the Principles of the Declaration of Istanbul as outlined in the Declaration of Istanbul on Organ Trafficking and Transplant Tourism. All patients gave their informed consent.

### Study protocol

GFR, serum creatinine, plasma BNP, plasma and urinary NGAL, plasma and urinary concentrations of LMWPs cystatin C and β2-microglobulin, urinary albumin excretion and urinary activities of tubular enzymes gamma-glutamyl transferase (GGT), lactate dehydrogenase (LDH), and *N*-acetyl-β-d-glucosaminidase (NAG) were measured in the 310 CKD patients at the time of GFR measurement. Plasma BNP and NGAL, and serum creatinine, cystatin C and β2-microglobulin were measured in maintenance hemodialysis (MHD) patients before and at the end of a mid-week hemodialysis session. Serum and urinary samples were divided into Eppendorf tubes and stored at -20°C.

### Measurement of glomerular filtration rate (reference test)

GFR was measured as the renal clearance of ^99m^Tc-DTPA [12,13], and was scaled to the standard body surface of 1.73 m^2^. Patients were assigned to CKD stages on the basis of the value of the measured GFR: CKD stage 1, GFR >90 ml/minute/1.73 m^2^; CKD stage 2, GFR 60 to 90 ml/minute/1.73 m^2^; CKD stage 3a, GFR 45 to 60 ml/minute/1.73 m^2^; CKD stage 3b, GFR 30 to 45 ml/minute/1.73 m^2^; CKD stage 4, GFR 15 to 30 ml/minute/1.73 m^2^; CKD stage 5, GFR <15 ml/minute/1.73 m^2^[11].

### Measurement of blood and urinary concentrations of NGAL, creatinine, cystatin C and β2-microglobulin, of plasma BNP, and of urinary excretion of albumin and tubular enzymes (index tests)

The Alere Triage® System (Alere San Diego, Inc., San Diego, CA, USA ) was used to determine plasma NGAL (Alere Triage® NGAL Test). The measurement utilizes antibodies with low affinity to the dimeric forms of NGAL. The upper limit of the reference range is 153 ng/ml (90% confidence interval, 142 to 182 ng/ml) (Triage® NGAL Product Insert).

To measure urine NGAL, an antibody sandwich immunoassay was utilized for urine samples. Test samples were added to a 384-well plate, containing a calibration curve and control samples. The plates were read by a fluorometer. The upper limits of the reference ranges reported for urine NGAL are 131.7 ng/ml (Urine NGAL Abbott, Product Insert; Abbott Ireland, Longford, Ireland), and 107 μg/l (males, 91 μg/l; females, 129 μg/l) [14].

Fractional excretion (FE) of NGAL was calculated as:

$$\begin{array}{cc} \mathrm{FE}=100\times & \left(\mathrm{urinary}\;\mathrm{NGAL}\times \mathrm{serum}\;\mathrm{creatinine}\right)\\ & /\left(\mathrm{plasma}\;\mathrm{NGAL}\times \mathrm{urinary}\;\mathrm{creatinine}\right) \end{array}$$

Plasma BNP was measured with the Alere Triage® System, utilizing antibodies against BNP (Alere Triage® BNP Test). The upper limit of the reference range is 100 pg/ml.

The average coefficients of variation for plasma NGAL and BNP, measured by means of the Alere Triage® System from two replicates of plasma specimens at four analyte levels tested at two separate times daily for 20 days, ranged between 9.2 and 12.8% for BNP and between 12.5 and 16.0% for NGAL (Triage® CardioRenal Panel Product Insert, 2011).

Creatinine was measured with a rate-blanked creatinine/Jaffé method (CREA Roche/Hitachi for Hitachi 917; Roche Diagnostics, Mannheim, Germany). The reference intervals for serum concentration were 0.50 to 0.90 mg/dl in females and 0.70 to 1.20 mg/dl in males.

Cystatin C was measured with a particle-enhanced immune-nephelometric method (N Latex Cystatin C; Dade Behring, Marburg, Germany). The reference intervals for serum concentrations were 0.53 to 0.95 mg/l, without differences between males and females. 

β2-Microglobulin was measured with an immune-enzymic method (AxSym β2-Microglobulin; Abbott, Wiesbaden, Germany). The mean reference value for serum concentration was 0.99 ± 0.16, without differences between males and females.

Urinary albumin was measured with an immune-nephelometric method (N antiserum to human albumin; Dade Behring, Marburg, Germany).

For measurement of urinary excretion of tubular enzymes, urinary activities of brush border enzyme GGT and cytosolic enzyme LDH were measured with methods routinely used for the determinations of these enzymes in plasma (Boehringer Mannheim for Hitachi 911; Boehringer Mannheim Biochemia, Basel, CH). Urinary activities of lysosomal enzyme NAG were measured by automating for Hitachi 911, a manual method (Boehringer Mannheim Biochemia).

FE of LMWPs was calculated with a formula similar to that for FE of NGAL. Since urinary NGAL is commonly expressed as μg/l, all other proteins and enzymes were expressed also as mg/l, mg/dl or u/l. The relative relationships are not affected if all data are normalized for urinary creatinine excretion.

### Statistical analysis

The reference intervals of all examined index tests have been calculated using a nonparametric method, as recommended by the Clinical and Laboratory Standards Institute [15]. These reference intervals take the central 95% of our reference population; that is, the 33 potential kidney donors (eight males, 25 females; age 25 to 66, mean 50.2 years). Note the high prevalence of women among kidney donors, and the difference in age distribution in comparison with the examined patients.

The correlation coefficients between GFR and the index tests were measured after logarithmic transformation of data. The significance of the differences among correlation coefficients was tested [16]. The significance of the differences between two independent samples and between two paired samples was tested using the nonparametric Mann–Whitney and Wilcoxon tests, respectively. The diagnostic accuracy of markers was assessed using receiver-operating characteristic analysis. Statistical analysis was performed using MedCalc (version 12.4.0.0; MedCalc Software, Mariakerke, Belgium). *P* < 0.05 was considered significant.

## Results

**Figure 1:**
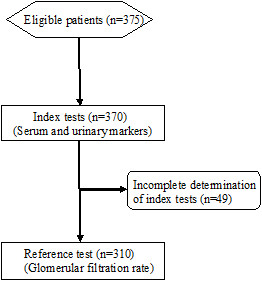
Flow diagram of the chronic kidney disease patients that entered the study.

The flow diagram of the 375 eligible patients, according to the STARD initiative [17], is reported in Figure 1. Fifty-four patients had inadequate collection of blood or urine samples for the determination of index tests. The measurement of GFR (reference test) was inadequate in 11 patients. The data for the remaining 310 CKD patients and 31 MHD patients are analyzed in the present study. Their main anthropometric and clinical data are presented in Table 1.

**Table 1:** Demographic and clinical data for chronic kidney disease patients and maintenance hemodialysis patients

	**Chronic kidney disease patients**		**Maintenance hemodialysis patients**	
	**Range**	**Mean ± SD**	**Range**	**Mean ± SD**
Age (years)	17 to 82	50.5 ± 14.2	36 to 92	69.9 ± 15.1
Body weight (kg)	34 to 119.4	73.4 ± 15.2	33 to 122	68.6 ± 18.9
Height (cm)	139 to 191	166 ± 9.4	148 to 182	167 ± 9.9
Body mass index (kg/m^2^)	13 to 42.8	26.5 ± 4.7	15 to 39	24.2 ± 5.8
Serum creatinine (mg/dl)	0.5 to 7.5	1.7 ± 1.2	2.4 to 17.0	8.6 ± 3.2
Native kidney disease (*n*)				
Chronic nephropathies	63		2	
Chronic glomerulonephritis	50		7	
Renal transplant recipient	42			
Diabetic nephropathy	36		6	
Ischemic nephropathy	15		10	
Oncological diseases	12			
Interstitial nephritis	8		3	
APKD	6		3	
Potential kidney donor	33			
Living kidney donor	45			

Chronic kidney disease patients: 161 males, 149 females; maintenance hemodialysis patients: 23 males, eight females. APKD, autosomal dominant polycystic kidney disease; SD, standard deviation.

The dialysis vintage of the 31 MHD patients ranged between 0.2 and 17 years (mean 3.5 ± 4.2 years). All patients were treated with a schedule of three sessions/week, mean length 4.0 ± 0.2 hours. Mean blood flow was 312 ± 42 ml/minute, and dialysate flow was fixed at 700 ml/minute. Twenty-three hemodialysis treatments were performed using a low-flux membrane (polysulfone), and 21 treatments were performed with high-flux membranes (cellulose triacetate or acrylonitrile or sodium methallyl sulfonate copolymer).

### Reference ranges

The reference intervals determined in the potential kidney donors were: plasma NGAL, 20 to 133 ng/ml; plasma BNP, 5 to 47 pg/ml; serum creatinine, 0.6 to 1.3 mg/dl; serum cystatin C, 0.4 to 1.06 mg/l; serum β2-microglobulin, 0.7 to 1.8 mg/l; urinary NGAL, 0 to 33 ng/ml; urinary albumin, 0.1 to 9.3 mg/dl; urinary cystatin C, 0 to 0.4 mg/l; urinary β2-microglobulin, 0.1 to 0.29 mg/l; GGT, 5 to 183 u/l; LDH, 0.1 to 86 u/l; and NAG, 1 to 16 u/l.

### Correlation of markers and chronic kidney disease stage

#### Effect of glomerular filtration rate impairment on NGAL and BNP

Plasma NGAL increased with the reduction of GFR (Table 2). A significant increase in mean values was already found at CKD stage 2. Plasma NGAL then progressively increased with the reduction of GFR and was even higher in MHD patients, up to 16 times the values at CKD stage 1. The mean values of serum creatinine, cystatin C, and β2-microglobulin also progressively increased with the stage of CKD. Only modest differences were found for BNP in the different CKD stages, while BNP was markedly increased in MHD patients. Urinary NGAL was significantly increased only in patients at CKD stages 4 and 5. A significant increase in mean values of urinary cystatin C was found starting from CKD stage 4, while urinary β2-microglobulin was significantly higher already at CKD stage 3a. Urinary excretion of LDH and NAG were similar at all stages of CKD, while values of GGT decreased slightly but significantly with the impairment of GFR, starting from CKD stage 2.

**Table 2:** Plasma and urinary concentrations of NGAL and BNP in comparison with serum and urinary concentrations of various markers of GFR impairment and urinary excretion of tubular enzymes

	**CKD stage 1**	**CKD stage 2**	**CKD stage 3a**	**CKD stage 3b**	**CKD stage 4**	**CKD stage 5**	**MHD**
Number	48	85	64	50	43	20	31
Age (years)	44.3 ± 2.1	49.1 ± 1.4	49.2 ± 1.7	52.1 ± 2.3*	58.3 ± 2.2****	54.6 ± 2.4**	69.9 ± 2.8****
GFR (ml/minute/1.73 m^2^)	111.3 ± 2.5	72.4 ± 1.0****	52.9 ± 0.5****	38.1 ± 0.6****	21.6 ± 0.6****	11.0 ± 0.6****	
Plasma NGAL (ng/ml)	66 ± 6.4	114 ± 10***	186 ± 18****	273 ± 26****	559 ± 59****	862 ± 95****	1093 ± 56****
Plasma BNP (pg/ml)	20 ± 4.3	24 ± 3.4	40 ± 7.2*	32 ± 5.4	62.9 ± 13**	86 ± 45	1116 ± 248
Serum creatinine (mg/dl)	0.8 ± 0.03	1.1 ± 0.03****	1.4 ± 0.15****	1.8 ± 0.17****	3.0 ± 0.16****	5.1 ± 0.28****	8.6 ± 0.6****
Serum cystatin C (mg/l)	0.8 ± 0.03	1.1 ± 0.03****	1.3 ± 0.04****	1.7 ± 0.06****	2.5 ± 0.11****	3.9 ± 0.26****	5.2 ± 0.2****
Serum β2-microglobulin (mg/l)	1.3 ± 0.06	1.8 ± 0.06****	2.3 ± 0.10****	3.3 ± 0.18****	5.8 ± 0.33****	9.5 ± 0.71****	32.3 ± 1.6****
Urinary NGAL (ng/ml)	13 ± 3	11 ± 2	15 ± 4	21 ± 6	38 ± 10*	90 ± 24**	
Urinary albumin (mg/dl)	16 ± 8.4	39 ± 18.9	38 ± 11.3	85 ± 31.9*	69 ± 11.4***	136 ± 64.5	
Urinary cystatin C (mg/l)	0.1 ± 0.02	0.1 ± 0.03	0.2 ± 0.08	0.2 ± 0.08	0.4 ± 0.10**	2.9 ± 1.15	
Urinary β2-microglobulin (mg/l)	0.3 ± 0.13	0.3 ± 0.12	0.8 ± 0.26*	1.7 ± 0.66**	4.7 ± 1.14***	19 ± 4.6**	
Urinary GGT (u/l)	71 ± 7.0	49 ± 4.3**	36 ± 3.9***	46 ± 5.9**	44 ± 9.1*	35 ± 7.8***	
Urinary LDH (u/l)	31 ± 3.7	32 ± 4.2	25 ± 6.0	28 ± 4.3	24 ± 3.6	67 ± 32.6	
Urinary NAG (u/l)	6.1 ± 0.5	6.3 ± 0.5	5.4 ± 0.4	6.2 ± 0.5	7.3 ± 0.9	5.6 ± 0.7	

Mean values ± standard errors of the mean are reported. The significance of the differences with the mean values of the group of patients at CKD stage 1 is indicated: *P < 0.05; **P < 0.01; ***P < 0.001; ***P < 0.0001. In MHD patients, plasma NGAL, BNP, serum creatinine, cystatin C and β2-microglobulin were measured immediately before the beginning of the dialysis session. BNP, B-type natriuretic peptide; CKD, chronic kidney disease; GGT, gamma-glutamyl-transferase; GFR, glomerular filtration rate; LDH, lactate dehydrogenase; NAG, *N*-acetyl-β-d-glucosaminidase; MHD, maintenance hemodialysis patients; NGAL, neutrophil gelatinase-associated lipocalin.

**Figure 2:**
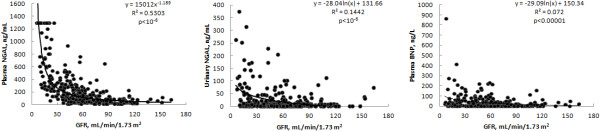
**Correlations with glomerular filtration rate of plasma and urinary neutrophil gelatinase**-**associated lipocalin and plasma B-type natriuretic peptide.** Regression lines, regression equations, coefficients of determination *R*^2^, and statistical significance of the correlations are reported. BNP, B-type natriuretic peptide; GFR, glomerular filtration rate; NGAL, neutrophil gelatinase-associated lipocalin.

The data for individual patients (Figure 2) indicate that plasma NGAL markedly and progressively increased, like serum creatinine, cystatin C and β2-microglobulin, with the reduction of GFR (Table 2). The correlation between plasma NGAL and these markers was highly significant (*R* = 0.745 with creatinine; *R* = 0.731 with cystatin C; *R* = 0.773 with β2-microglobulin). However, the correlation of plasma NGAL with GFR was significantly lower (*P* < 0.0001) than that of serum creatinine, cystatin C and β2-microglobulin. Plasma BNP increased slightly with the reduction of GFR (Figure 2) and its correlation with GFR was quite weak. Urinary NGAL significantly increased with the reduction of GFR, with a behavior similar to urinary cystatin C and β2-microglobulin (Figure 2, Table 2). Albuminuria had a weak but statistically significant correlation (*r* = 0.338, *P* < 0.001) with urinary NGAL.

The accuracy of plasma NGAL as an indicator of GFR impairment was significantly lower than that of serum creatinine, cystatin C and β2-microglobulin. The accuracy of urine NGAL and plasma BNP as indicators of GFR impairment was quite modest (Table 3).

**Table 3:** Accuracy of blood and urinary concentrations of the different markers as indicators of different degrees of glomerular filtration rate impairment evaluated by means of receiver operating curves

	**GFR < 90 ml/minute/1.73 m**^**2**^	**GFR <60 ml/minute/1.73 m**^**2**^	**GFR <45 ml/minute/1.73 m**^**2**^	**GFR <30 ml/minute/1.73 m**^**2**^	**GFR <15 ml/minute/1.73 m**^**2**^
Plasma NGAL (ng/ml)					
AUC	0.838*****	0.845*****	0.872*****	0.912*****	0.929*****
Criterion	>102	>136	>213	>214	>270
Sensitivity	69.8	77.4	77.0	92.1	100
Specificity	87.5	82.0	84.3	76.1	75.5
Plasma BNP (pg/ml)					
AUC	0.613**	0.599**	0.588*	0.623**	0.579 NS
Criterion	>31.3	>51.9	>26.6	>26.6	>28.3
Sensitivity	34	25	48	54	50
Specificity	85	93	71	68	67
Serum creatinine (mg/dl)					
AUC	0.895*****	0.908*****	0.943*****	0.977*****	0.990*****
Criterion	>1.01	>1.25	>1.55	>2.14	>3.08
Sensitivity	82.9	81.0	85.6	90.2	100
Specificity	85.6	81.7	89.2	96.7	94
Urinary NGAL (ng/ml)					
AUC	0.555 NS	0.601**	0.617***	0.635****	0.773***
Criterion		>8.2	>14.4	>20.2	>22.0
Sensitivity		49	42	47.6	70
Specificity		68	80	83	81

Values of the areas under the curve (AUC), criterion values, and sensitivity and specificity values are reported. The statistical significance of the different AUCs is indicated. *P < 0.05; **P < 0.01; ***P < 0.001;****P < 0.00001. BNP, B-type natriuretic peptide; GFR, glomerular filtration rate; NGAL, neutrophil gelatinase-associated lipocalin.

**Figure 3:**
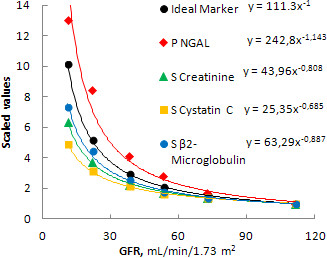
**Plasma concentrations of the various markers in comparison with an ideal marker of glomerular filtration rate impairment.** Patients are clustered in groups according to chronic kidney disease (CKD) stage. Values are scaled on the mean values of CKD patients at stage 1. GFR, glomerular filtration rate; NGAL, neutrophil gelatinase-associated lipocalin; P, plasma; S, serum.

The mathematic function that expresses the relationship between the ideal marker and GFR is an equilateral hyperbole. The curve of the ideal marker was generated assuming arbitrarily as 1 its plasma level at the value of GFR measured in CKD patients at stage 1 (111.3 ml/minute/1.73 m^2^). The relationships of various serum markers with GFR were different (Figure 3). In fact, while plasma β2-microglobulin, creatinine and cystatin C increased less than expected relative to GFR, plasma NGAL increased more than expected. Furthermore, the correlation among the various serum markers was different in the different CKD stages (Table 2, Figure 3). In particular, in CKD stage 2 the increases of serum creatinine, cystatin C and β2-microglobulin were identical (1.4 times the values measured in patients at CKD stage 1). Starting from CKD stage 3a the serum cystatin C increased less than creatinine and β2-microglobulin (1.6 versus times), and from stage 3b serum creatinine also increased less than β2-microglobulin (2.2 versus 2.5 times). These differences increased with the worsening of renal function: at CKD stage 5 the increases of serum cystatin C, creatinine and β2-microglobulin were 4.9, 6.4 and 7.3 times the values in patients at CKD stage 1.

The specificity of the various markers was also affected differently by GFR impairment (Table 4). A relevant number of false positive values of plasma NGAL was found already at CKD stages 2 and 3a, and at CKD stage 5 the percentage of false positive values was 100%. The false positive results of urinary NGAL were low at early CKD stages, but progressively increased up to 40% at CKD stage 5. Plasma BNP presented false positive results in a low percentage of CKD patients.

**Table 4:** Percentage of patients with values of the markers above the upper limit of the reference ranges in the different functional stages of chronic kidney disease

**CKD stage**	***N***	**Plasma NGAL >153 ng/ml ( *n */ *N *)**	**Percentage**	**Urine NGAL >131.7 ng/ml ( *n */ *N *)**	**Percentage**	**Plasma BNP >100 g/ml ( *n */ *N *)**	**Percentage**
1	48	1/48	2	2/48	4	2/48	4
2	85	18/85	21	1/85	1	2/85	2
3a	64	31/64	48	3/64	5	7/64	11
3b	50	36/50	72	3/50	6	4/50	8
4	43	39/43	91	5/43	12	9/43	21
5	20	20/20	100	8/20	40	3/20	15
Overall	310	145/310	47	22/310	7	27/310	9

BNP, B-type natriuretic peptide; CKD, chronic kidney disease; NGAL, neutrophil gelatinase-associated lipocalin.

**Figure 4:**
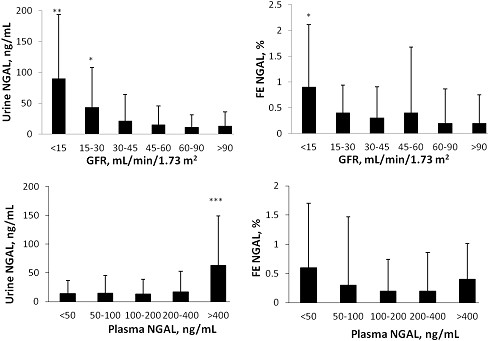
**Urinary concentration and fractional excretion of neutrophil gelatinase-associated lipocalin.** Urinary concentration and fractional excretion of neutrophil gelatinase-associated lipocalin (NGAL) in patients clustered in groups according to glomerular filtration rate (GFR; upper part) or according to plasma concentrations (lower part). Mean values and standard deviations are reported. The statistical significance of the differences versus the mean values in patients with GFR >90 ml/minute/1.73 m^2^ and versus the mean values in patients with the lowest blood concentrations of plasma NGAL are reported. *P < 0.05.

The urinary concentration and FE of NGAL were significantly increased at CKD stages 4 and 5, respectively (Figure 4). Urinary concentration of NGAL, but not FE of NGAL, also significantly increased when plasma NGAL was higher than 400 ng/ml. Urinary concentrations and FE of cystatin C and β2-microglobulin increased to a higher extent than NGAL with the reduction of GFR, and with the increase in their plasma levels.

Multiple regression analysis demonstrated that urinary NGAL is significantly correlated with plasma NGAL (*P* < 0.0001), with urinary β2-microglobulin (*P* < 0.0001), and with urinary albumin concentration (*P* = 0.005). In patients with urinary protein concentration <200 mg/l (mean 35 ± 43), urinary NGAL was 13 ± 26 ng/ml; while in patients with urinary albumin >2 g/l (mean 4.7 ± 4.0), urinary NGAL was significantly higher (41 ± 71 ng/ml; *P* < 0.001)

### Effect of native kidney disease on NGAL and BNP

Plasma NGAL values were significantly higher in kidney donors (post nephrectomy) than in potential kidney donors (Table 5). Plasma NGAL levels were also significantly higher in diabetics and in chronic glomerulonephrites than in kidney donors, even if the three groups of patients had similar values of GFR. Plasma BNP was also affected by the cause of renal disease. In fact, BNP was significantly higher in renal transplant recipients and in diabetics than in kidney donors with similar levels of GFR. On the contrary, urinary NGAL showed only slight differences according to the cause of kidney disease. Urinary excretion of tubular enzymes NAG and LDH were similar in all patients, while GGT modestly, but significantly, decreased with the reduction in GFR.

**Table 5:** Plasma and urinary concentrations of NGAL, BNP and, for comparison, serum and urinary concentrations of various markers of GFR impairment and urinary excretion of tubular enzymes, according to the etiologies of kidney disease

	**Potential kidney donors**	**Kidney donors**	**Renal transplant recipients**	**Chronic glomerulonephritis**	**Chronic kidney disease**	**Ischemic interstitial nephropathy**	**Diabetic nephropathy**
Number	33	45	42	50	63	29	36
Age (years)	50.2 ± 1.6	53.0 ± 1.6	41.8 ± 1.7***^++++^	43.2 ± 2.2*	55.5 ± 1.7*	58.1 ± 2.8*	49.9 ± 2.7
GFR (ml/minute/1.73 m^2^)	96.5 ± 3.6	62.4 ± 2.5****	47.4 ± 2.3****^++++^	58.6 ± 4.2****	38.3 ± 3.3****^++++^	43.5 ± 5.7****^++^	65.1 ± 6.5**^++^
Plasma NGAL (ng/ml)	55.9 ± 5.3	112.4 ± 11.3****	242.6 ± 34.6****^+++^	272.9 ± 50.1****^++^	456.8 ± 50.87****^++++^	371.1 ± 64.9****^+++^	215.6 ± 31.0****^++^
Plasma BNP (pg/ml)	19.1 ± 4.4	20.2 ± 0.9	56 ± 12.3**^++^	16.6 ± 3.1	35.9 ± 6.4**^++^	79.3 ± 31.6	48.2 ± 8.8**^++^
Serum creatinine (mg/dl)	0.8 ± 0.03	1.2 ± 0.04****	1.7 ± 0.1****^++++^	1.8 ± 0.2****^++^	2.6 ± 0.2****^++++^	2.2 ± 0.3***^++^	1.6 ± 0.13**^+^
Serum cystatin C (mg/l)	0.7 ± 0.03	1.1 ± 0.11****	1.6 ± 0.1****^++++^	1.6 ± 0.1****^++^	2.1 ± 0.1****^++++^	2.1 ± 0.3****^+++^	1.5 ± 0.1***^+^
Serum β2-microglobulin (mg/l)	1.2 ± 0.04	1.9 ± 0.04****	3.0 ± 0.3****^++^	3.1 ± 0.4****^++^	4.8 ± 0.4****^++++^	4.4 ± 0.7***^++^	2.8 ± 0.3***^++^
Urinary NGAL (ng/ml)	12.4 ± 4.6	10.1 ± 2.7	21.1 ± 7.0	27.3 ± 7.2^+^	45.3 ± 9.9**^+++^	17.4 ± 5.3	13.8 ± 3.4
Urinary albumin (mg/dl)	6.3 ± 4.3	1.6 ± 0.4	16.4 ± 8.4	184 ± 49***^+++^	52.9 ± 8.4****^++++^	60.0 ± 18.3**^++^	36.2 ± 11.2*
Urinary cystatin C (mg/l)	0.1 ± 0.07	0.1 ± 0.04	0.2 ± 0.09	0.9 ± 0.47	0.6 ± 0.16*^++^	0.4 ± 0.14	0.1 ± 0.04
Urinary β2-microglobulin (mg/l)	0.1 ± 0.06	0.3 ± 0.08*	1.7 ± 0.5***^++^	3.7 ± 1.6*^+^	5.2 ± 1.5***^++^	3.1 ± 1.4*^+^	1.1 ± 0.3*
Urinary GGT (u/l)	68.7 ± 9.5	29.0 ± 2.7***	40.6 ± 6.4*	63.6 ± 6.0^++++^	38.0 ± 4.5**	47.0 ± 9.1	43.3 ± 6.7**
Urinary LDH (u/l)	29.0 ± 4.1	18.3 ± 2.6*	17.3 ± 2.0*	51.8 ± 12.6^+^	25.0 ± 2.4^+^	47.3 ± 19.1	31.2 ± 6.5
Urinary NAG (u/l)	5.3 ± 0.6	5.1 ± 0.8	5.6 ± 0.5	6.8 ± 0.6^+^	7.1 ± 0.6*^++^	7.0 ± 0.9	5.7 ± 0.8

Mean values ± standard errors of the mean are reported. The significance of the differences with mean values of potential kidney donors and of kidney donors is indicated: *P < 0.05; **P < 0.01; ***P < 0.001; ****P < 0.0001; ^+^*P* < 0.05; ^++^*P* < 0.01; ^+++^*P* < 0.001; ^+++^*P* < 0.0001. BNP, B-type natriuretic peptide; GGT, gamma-glutamyl-transferase; GFR, glomerular filtration rate; LDH, lactate dehydrogenase; NAG, *N*-acetyl-β-d-glucosaminidase; NGAL, neutrophil gelatinase-associated lipocalin.

### Maintenance hemodialysis patients

**Figure 5:**
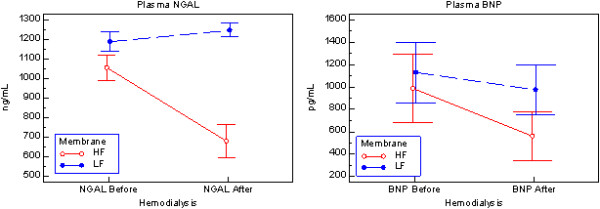
**Plasma concentrations of neutrophil gelatinase-associated lipocalin and B-type natriuretic peptide before and after a dialysis session with high-flux or low-flux membranes.** Plasma concentrations of neutrophil gelatinase-associated lipocalin (NGAL; left side) and B-type natriuretic peptide (BNP; right side) before and after a dialysis session with high-flux (HF; ○) membrane in 21 patients or with low-flux (LF; ●) membranes in 23 patients. Mean values ± standard error of the mean are reported.

In MHD patients, dialysis with high-flux membranes decreased plasma NGAL by 35.5% (*P* < 0.0001), while low-flux membranes did not remove plasma NGAL. Plasma BNP was also markedly decreased (-43.1%, *P* = 0.0005) after dialysis with high-flux membranes, more than with low-flux membranes (-13.8%, *P =* NS) (Figure 5).

## Discussion

NGAL is a LMWP (molecular weight (MW) 25 kDa) of the lipocalin family, which is constitutively and/or inducibly expressed in different tissues and cells [18,19]. NGAL is cleared from the plasma mainly through glomerular filtration. After filtration, NGAL is avidly reabsorbed into proximal tubular cells by endocytosis via the megalin system [20]. AKI upregulates NGAL mRNA in distal renal tubules, and also increases the production of NGAL by the lung, liver, and leukocytes [21]. In AKI, NGAL arrives into the urine from specific cells of distal tubule where it is overexpressed during tubular stress [22,23]. Furthermore, when the proximal tubule is damaged by acute tubular necrosis, filtered NGAL can escape tubular reabsorption and is excreted with the urine. Finally, when high plasma NGAL and the consequent high filtered charge saturates the tubular reabsorption capacity, the urinary excretion of NGAL could also increase, similar to other LMWPs [24].

Plasma and urinary concentrations of NGAL have been indicated as sensitive markers of AKI, in different clinical settings. A meta-analysis of data from 19 studies indicates that both plasma/serum and urine NGAL appear to perform similarly well for diagnostic and prognostic evaluation of AKI [25].

BNP is a LMWP (MW 6.5 kDa) that belongs to the family of natriuretic peptides. It is produced by enzymatic cleavage of pro-BNP, which is secreted into the plasma from the myocardial cells in patients with left ventricular hypertrophy and/or dysfunction, or fluid overload. Plasma BNP can assess the occurrence, severity and prognosis of acute and chronic heart failure. BNP is cleared from the plasma not only by the kidneys, via glomerular filtration and tubular catabolism, but also due to its cleavage by proteolytic enzymes.

The concurrent cardio-renal assessment, combining the measurement of BNP and NGAL, has been proposed as a powerful predictor of AKI [26].

Similarly to other LMWPs cleared by the plasma via glomerular filtration, one can expect plasma concentrations of NGAL and BNP to also increase when GFR decreases. Indeed, it is known that plasma BNP increases in heart failure patients with decreased renal function, in CKD patients and, markedly, in MHD patients [7,8]. Different studies in CKD patients, renal transplant recipients, and diabetics suggest that plasma and/or urine NGAL are correlated with serum creatinine and with predicted GFR, or that NGAL may predict the worsening of renal function [27-31]. On the other hand, NGAL was not a reliable predictor of worsening of diabetic nephropathy [32,33]. In renal transplant recipients, some concern has been raised about the usefulness of NGAL as a screening test for renal function impairment [34]. After heart and lung transplantation, plasma NGAL was also not correlated with cystatin C and estimated GFR [35]. Finally, only urine NGAL was found to be correlated with CKD in type 2 diabetics with nephropathy and in pediatric heart transplant recipients [36,37]. These literature data indicate the need for a study involving CKD patients at various functional stages with stable renal function, to evaluate the relationship of plasma and urine concentrations of NGAL with measured GFR.

The present study was performed in CKD patients affected by different kidney diseases, with stable renal function, and various degrees of GFR impairment, from normality to advanced renal failure, to assess the relationship of NGAL and BNP with measured GFR. To clarify both glomerular and tubular mechanisms of renal handling of NGAL, plasma and urinary concentrations of the LMWPs cystatin C (MW 13.4 kDa) and β2-microglobulin (MW 11.8 kDa), and urinary excretion of tubular enzymes were also measured. The reference intervals of the different index tests, measured in potential kidney donors, agreed with data from the literature. However, note that the modest number of subjects and their gender and age distribution do not allow one to consider the results obtained to be true reference intervals.

The major result of this study is that, in CKD patients with stable renal function, plasma NGAL concentration increases progressively with the reduction of GFR, according to an exponential function similar to those of serum markers of GFR impairment. These results demonstrate that plasma removal of NGAL is greatly impaired by the decrease of GFR, reducing the accuracy of NGAL as a marker of AKI. In fact, increased plasma values of NGAL, suggestive for AKI, were found in a high percentage of CKD patients with stably impaired renal function. The percentage of false positive NGAL increased with the stage of CKD, up to 100% at CKD stage 5. Other literature data indicate that plasma NGAL levels do not adequately predict AKI in patients with acute heart failure, and were significantly correlated with serum creatinine values at presentation [38]. According to a recent study, the choice of a higher threshold (plasma NGAL >400 ng/ml) increases the specificity for the diagnosis of AKI among emergency department patients, with a pre-existing CKD [39].

Furthermore, in the present study, the increase in plasma NGAL concentration found with decreasing GFR was too high to consider NGAL an ideal marker of GFR, suggesting that NGAL production increases in renal failure. Hypothetically, similar results could also be due the interference of increased plasma values of homodimer NGAL, which due to its MW of ~50 kDa has a low filtration coefficient [40].

Our study also demonstrated that urinary NGAL and plasma BNP increase significantly with the reduction of GFR. However, urinary NGAL is less affected than plasma NGAL by the level of GFR and its reabsorption by the proximal tubule is not easily saturated. In fact, urinary NGAL increased only at CKD stage 4 and when the plasma concentration of NGAL was quite high (>400 ng/ml), while its FE remained quite low, lower than those of the two other LMWPs cystatin C and β2-microglobulin. The effect of the reduction of GFR was even lower on plasma BNP, which, possibly due to its extra-renal clearance, increased markedly only in MHD patients, as reported previously [7,8]. The relationships between urinary NGAL and urinary albumin and β2-microglobulin suggest a possible competition for tubular reabsorption of NGAL with albumin and β2-microglobulin. These data are in agreement with animal data demonstrating that proteinuria was associated with increasing urinary NGAL concentrations [41]. Further studies are needed to establish the necessity of different reference ranges for urinary NGAL in patients with proteinuria.

A clear difference is evident in the relationship with GFR between urinary excretion of functional and injury markers. In fact, urinary excretions of functional markers (cystatin C and β2-microglobulin) increase with the reduction of GFR, due to the saturation of their tubular reabsorption. To the contrary, injury markers (LDH and NAG) are not affected by the decrease of GFR, or eventually the urinary excretion (GGT) may decrease with the reduction of GFR, probably due to the reduction of renal mass. The behavior of NGAL appears similar to that of functional markers.

The increase in plasma concentration and urinary excretion of NGAL that accompanies the decrease of GFR may be determined by an increase of its extra-renal production, justified by the proinflammatory status of CKD and/or by its increased production by inflamed tubular cells in CKD patients, as suggested by the forest fire theory proposed by Mori and Nakao [42]. The different effect of the various underlying kidney diseases on plasma and urine NGAL concentrations is in agreement with this theory. On the other hand, the doubling of plasma NGAL in healthy living kidney donors, which is not accompanied by an increase in urinary NGAL or in tubular enzymes, indicates a significant effect of the reduction of GFR *per se*. Finally, the relevance of the level of GFR on plasma NGAL concentration is clearly indicated by the extremely high plasma NGAL measured in MHD patients with no residual renal function. In MHD only hemodialysis with high-flux membranes removes plasma NGAL, due to its quite high MW. Thus, it is important to consider that the treatment of severe AKI by hemodialysis can mask a persistent increase in plasma NGAL. In MHD patients, plasma BNP concentrations are lowered by both low-flux and high-flux hemodialysis, in agreement with literature data [7,8,43-45]. The possible removal of BNP by dialysis must therefore be considered when evaluating the effect of HD treatment on heart failure.

## Conclusions

Plasma NGAL increases markedly with a reduction in GFR in stable CKD patients, potentially producing a very high number of false positive diagnoses for AKI. GFR impairment and the etiology of kidney disease have a more relevant effect on plasma NGAL than on urinary NGAL. Thus, urine NGAL should be more accurate than plasma NGAL as a marker of AKI in CKD patients. In any case, specific reference values of NGAL and BNP should be used in CKD patients, according to the CKD stage.

## Key messages

• The impairment of GFR in CKD patients affects mainly plasma NGAL, and to a lower extent urinary NGAL and plasma BNP.

• The increased values of plasma NGAL could determine a high number of false positive diagnoses for AKI in stable CKD patients.

• Specific reference ranges for NGAL and BNP should be used in CKD patients, according to the CKD stage.

## Abbreviations

AKI: acute kidney injury; BNP: B-type natriuretic peptide; CKD: chronic kidney disease; FE: fractional excretion; GGT: gamma-glutamyl transferase; GFR: glomerular filtration rate; LDH: lactate dehydrogenase; LMWP: low-molecular weight protein; MHD: maintenance hemodialysis patients; MW: molecular weight; NAG: *N*-acetyl-β-d-glucosaminidase; NGAL: neutrophil gelatinase-associated lipocalin.

## Competing interests

The author declares that he has no competing interests.
